# Seed dormancy-breaking in a cold desert shrub in relation to sand temperature and moisture

**DOI:** 10.1093/aobpla/plx003

**Published:** 2017-01-25

**Authors:** Huiliang Liu, Buhailiqiemu Abudureheman, Lingwei Zhang, Jerry M. Baskin, Carol C. Baskin, Daoyuan Zhang

**Affiliations:** 1 Key Laboratory of Biogeography and Bioresource in Arid Land, Xinjiang Institute of Ecology and Geography, Chinese Academy of Sciences, Urümqi 830011, China; 2 Turpan Eremophytes Botanical Garden, Chinese Academy of Sciences, Turpan 838008, China; 3 Xinjiang Institute of Science and Technology (Aksu Campus), Aksu 843000, China; 4 College of Grassland and Environment Sciences, Xinjiang Agricultural University, Xinjiang Key Laboratory of Soil and Plant Ecological Processes, Urümqi 830052, China; 5 Department of Biology, University of Kentucky, Lexington, KY 40506, USA; 6 Department of Plant and Soil Sciences, University of Kentucky, Lexington, KY 40546, USA

**Keywords:** Burial depth, *Eremosparton songoricum*, Fabaceae, physical dormancy, seed germination phenology, wet-dry cycles

## Abstract

Seasonal periodicity of seed germination and its relationship to seasonal changes in temperature and soil moisture have been well studied in seeds of species with physiological dormancy. However, relatively little information is available on the role of these environmental factors in controlling germination of seeds with physical dormancy (PY). Our primary aim was to determine if seeds of the cold desert sand dune semi-shrub *Eremosparton songoricum* exhibits seasonal periodicity of seed germination and the relationship between seed dormancy break and soil temperature and moisture. In the laboratory, seeds incubated on dry, wet, wet-dry and dry-wet sand were exposed to a 1-year sequence of temperature regimes simulating those in the field. In the field, seeds were buried at different depths on a sand dune, and germination of periodically exhumed seeds was tested at five temperature regimes during a 2-year period. In the 1-year sequence of simulated natural temperature regimes, breaking of PY was more effective under constantly wet than under constantly dry conditions, and germination percentage was significantly higher under dry-wet than under wet-dry conditions. Seeds buried in the field exhibited a distinct peak of germination in spring and little or no germination in other seasons. The final (2-year) monthly cumulative germination percentage differed among burial depths and temperature, and it was highest (47 %) in seeds buried at 3 cm and tested at 25/10 °C. A seed cohort of *E. songoricum* likely exhibits a long-term annual periodicity of spring germination in the field, and dormancy break appears to be driven by low (winter) temperatures and relatively high sand moisture content. To our knowledge this is the first study to document seasonal periodicity in seed germination in a cold desert species with PY and to identify the mechanism (at the whole-seed level) of its occurrence.

## Teaser

Eremosparton songoricum (Litv.) Vass. (Fabaceae, Papilionoideae) is a perennial semi-shrub, inhabiting the cold desert. Freshly matured seeds of E. songoricum have physical dormancy (PY). In this study, we determined whether seeds of E. songoricum exhibit seasonal periodicity of seed germination and the relationship between seed dormancy break and soil temperature and moisture. To our knowledge this is the first study to document seasonal periodicity in seed germination in a cold desert species with PY and to identify the mechanism (at the whole-seed level) of its occurrence.

## Introduction

Physical dormancy (PY) in seeds is caused by a water impermeable seed or fruit coat, and thus seeds cannot imbibe water when placed in a hydrated environment (Jayasuriya *et al.* 2013; Baskin and Baskin 2014). PY is present in at least 18 families of angiosperms including many taxa of Fabaceae (Baskin and Baskin 2014). Studies on the ecological factors affecting PY release have shown that high temperatures and/or dry heat (McKeon and Mott 1982; Lonsdale 1993; Norman *et al.* 2002), alternate wetting and drying (Baskin and Baskin 1984), wet heat (Van Klinken and Flack 2005; Van Klinken *et al*. 2006) and low (5°C) temperature (Van Assche *et al.* 2003) are effective for dormancy-break, depending on the species. Thus, temperature and moisture are expected to be the two critical factors that affect PY release in nature.

Mature seeds with PY are dispersed from maternal plants and may become buried in the soil, where PY-break is controlled via soil temperature and moisture conditions (Hu *et al.* 2009; Baskin and Baskin 2014). As burial depth increases, the rate of PY release may decrease (Taylor and Ewing 1988; Van Klinken and Flack 2005; Van Klinken *et al.* 2006), increase (Hu *et al.* 2009) or increase and then decrease (Loi *et al.* 1999; Zeng *et al.* 2005). Burial depth is closely related to soil temperature and moisture. However, studies generally have focused on burial depth in relation to breaking of PY and have not been concerned with temperature or soil moisture (see Baskin and Baskin 2014).

In the cold deserts of northwestern China, temperature is high in summer and low in winter, and amount and timing of rainfall is irregular. Based on available information from other systems, we infer that temperature, moisture and burial in the natural habitat affect PY release in seeds of cold desert species. To explore the breaking of PY in the cold desert, we selected *Eremosparton songoricum* (Litv.) Vass. (Fabaceae, Papilionoideae) as our study organism. This species is a perennial semi-shrub that is endemic to fixed and semi-fixed sand dunes of the Gurbantunggut Desert of Xinjiang Autonomous Region of northwest China and inland sand dunes around Lake Balkash in Kazakhstan (Yin *et al.* 2006; Zhang *et al.* 2008). Flowering occurs from late spring to early summer, and fruits are mature by mid- to late summer (Liu *et al.* 2011, 2013). The aboveground stems die in winter, and new shoots emerge from buried rhizomes or aerial-exposed stems the following spring (Wang *et al.* 2011). The dispersal unit is a pod that contains one or two seeds inside a papery pericarp (Liu *et al.* 2011; Zhang *et al.* 2011a). Plants are self-compatible, but pollination success relies on pollinators visiting the flowers to aid release of pollen onto the stigma (Zhang *et al.* 2011b). The species does not produce seeds by apomixis (Shi *et al.* 2010a, *b*; Zhang *et al.* 2011a, *b*).

Freshly matured seeds of *E. songoricum* have PY, i.e. water-impermeable seed coat. The highest germination of intact *E. songoricum* seeds in a study by Liu *et al.* (2011) was only 9 %, whereas artificially scarified seeds germinated to 69.3 %. The pericarp of the fruit remains intact during winter, and seeds have been observed germinating in the field in spring within the pericarp (H.-L Liu, pers. obs.). In nature, seeds of *E. songoricum* are inevitably buried by moving sand (Liu *et al.* 2011). After 7 months of burial at a depth of 3 cm in the field, only 23 % of the seeds germinated when tested at 25/10°C (temperature regime simulating that in the field in late April and early May), indicating that most seeds in a cohort needed additional time to release PY under natural conditions. Further, there is little or no information on the temperature and moisture conditions required for release of PY in seeds of *E. songoricum* seeds under field conditions. Thus, the aims of our study were to (i) monitor the phenology of PY breaking of *E. songoricum* seeds in the field and thus determine if the species exhibits seasonal germination periodicity, and (ii) identify the relationship between dormancy-break and temperature and moisture conditions.

## Methods

### Seed collection

Mature fruits of *E. songoricum* were collected from several hundred plants growing on sand dunes near the Caiman oil station (45°00′78″N, 88°22′94″E, 627 m a.s.l.) in the Gurbantunggut Desert of China in September 2011 and kept for about 2 weeks in ambient laboratory conditions. Then, the seeds were manually removed from the fruits and intact ones stored in paper bags under ambient laboratory conditions until the start of experiments.

### Meteorological data

Based on data from the nearest weather station (Fukang Ecology Station, 2001–10), about 10 km from our study site, mean annual temperature is 8.3 °C, and mean temperature of the coldest (January) and hottest (July) months are −15.6 and 26.0 °C, respectively. Mean annual precipitation is 222 mm (including snow in winter), about two-thirds of which falls in spring and summer, and the snow that falls in winter begins to melt in March or April. Mean potential annual evaporation in the Junggar Basin, in which our study site and the Fukang Ecology Station are located, is >2000 mm (Wei *et al.* 2003).

We monitored soil temperature and moisture in the field (near Fukang Ecology Station) in a natural population of *E. songoricum* with a Soil Four Parameter Automatic Recorder at 1-h intervals, using a WatchDog Micro Station (Spectrum Technologies, Inc., IL, USA). Temperature was monitored on the bare sand surface and at 3 cm (a suitable depth for seedling emergence) and 8 cm (an unsuitable depth for seedling emergence) below the sand surface but moisture only at the two sand depths. The temperature sensor on the surface was covered with enough sand so that it was not visible; thus, it was not exposed to direct sun light.

### Effect of simulated natural temperature and moisture conditions on PY-break

**Figure 1.**
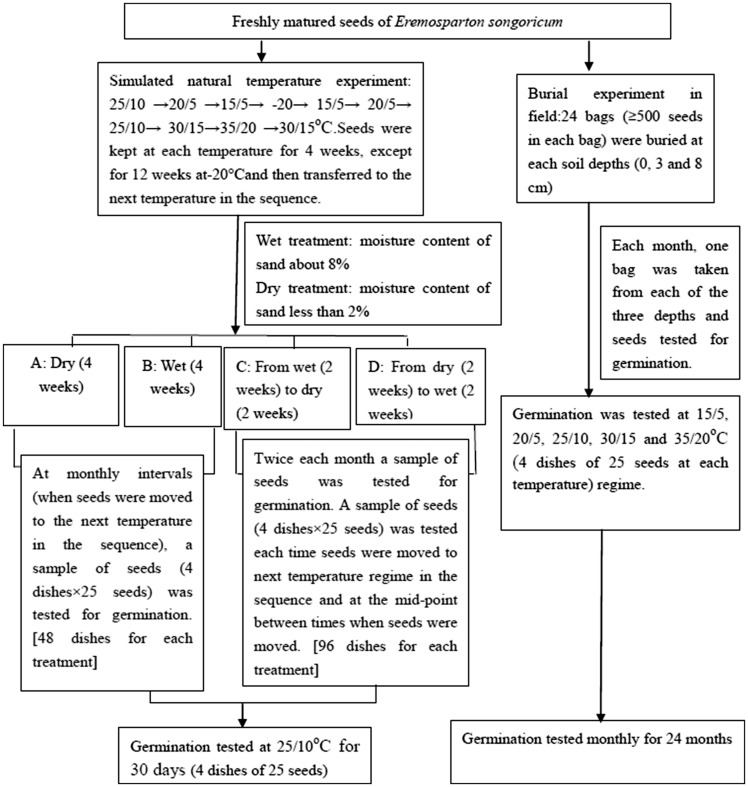
Experimental design of study on *E. songoricum*.

To determine the effect of temperature and moisture on the breaking of PY, seeds were subjected to a sequence of temperature and moisture conditions in the laboratory (Fig. 1). The temperature regimes simulate the average maximum and minimum monthly air temperatures in the natural habitat of *E. songoricum*. This experiment was started in September 2011 using mature seeds. Thus, the sequence of temperatures was 25/10°C (September) → 20/5°C (October) → 15/5 °C (November) →−20 °C (December, January, February) → 15/5 °C (March) → 20/5°C (April) → 25/10°C (May) → 30/15°C (June) → 35/20°C (July) → 30/15°C (August). Seeds were exposed to four moisture conditions during movement through the sequence of temperature regimes: A, constant dry; B, constant wet; C, alternate wet-dry; and D, alternate dry-wet. Forty eight dishes each were used for A: (dry) and for B: (wet) (4 dishes × 1 test/month × 12 months) and ninety six dishes each for C: and for D: (4 dishes × 2 tests/month × 12 months). Twenty-five fresh seeds were placed in each Petri dish, which was filled with sterilized dry or wet sand from the habitat, and the lids were sealed with Parafilm. In the wet treatment, distilled water was added once a week to keep the sand moist. For the wet-dry (C) and dry-wet (D) treatments, seeds were kept wet for 2 weeks and dry for 2 weeks each month. In the wet-dry treatment, the wet period was given first, and in the dry-wet treatment the dry period was given first. Distilled water was added once a week during the wet period, and no water was added during the dry period. At the end of the wet period, seeds were transferred to Petri dishes filled with dry sand and kept dry for 2 weeks. When seeds were at −20°C, the wet sand was frozen.

Germination trial was carried out each time seeds were moved to the next temperature in the sequence with four dishes of 25 seeds in light/dark (12 h/12 h) at 25/10 °C for 30 days (Fig. 1). The test temperature regime of 25/10 °C represents the spring germination season. The number of seeds that germinated while at a particular temperature in the sequence was added to the number that germinated when seeds were subsequently tested at 25/10 °C.

### Phenology of PY-break in the field

On 15 September 2011, 500 freshly matured seeds of *E. songoricum* were placed in each of 72 nylon-mesh bags (15 cm long × 10 cm wide). Twenty-four bags were placed at each soil depth (0, 3, 8 cm) near the Fukang Ecology Station. One bag each from the surface and from 3 and 8 cm sand depths was retrieved from the field on the first day of each month, beginning on 15 October 2011 and ending on 15 October 2013. Retrieved seeds were placed on filter paper moistened with distilled water in 9 cm diameter Petri dishes and incubated at 15/5, 20/5, 25/10, 30/15 and 35/20 °C (12-/12-h, light/dark). Four replicates of 25 seeds were used in each test condition and final germination percentages determined after 30 days. Two germinated seeds were found in the bags from 3 cm in May 2013; no other germinated seeds were found in the bags during the study. These numbers were added to final germination percentage at 25/10 °C.

### Statistical analysis

Means of germination percentages of seeds subjected to different moisture regimes and to different periods of exposure to the sequence of temperature regime in the laboratory experiment were compared using analyses of variance (Two-way ANOVAs). *Posthoc* Tukey test was performed for multiple comparisons to identify significant differences among treatments. General Linear Model (GLM) for univariate analyses was applied to identify possible effects of burial depths and temperatures regimes in germination trials (explanatory variables) on seed germination percentage (response variable). Pearson correlation analysis was used to identify the correlation of germination percentage after 2 years in the field with soil temperature and moisture. Partial correlation analyses of germination percentages after 2 years in the field, soil temperature and moisture allowed testing the correlation between two of these variables after removing the influence of a third variable. We controlled effect of soil temperature and soil moisture and performed this analysis for all burial depths and temperatures regimes of germination trials. Germination data in different moisture conditions and burial depths were arcsine square-root transformed when necessary to meet assumptions of ANOVA for normality and homogeneity of variance. All analyses were performed with SPSS v.16.0 at *α* = 0.05.

## Results

### Meteorological data

**Figure 2.**
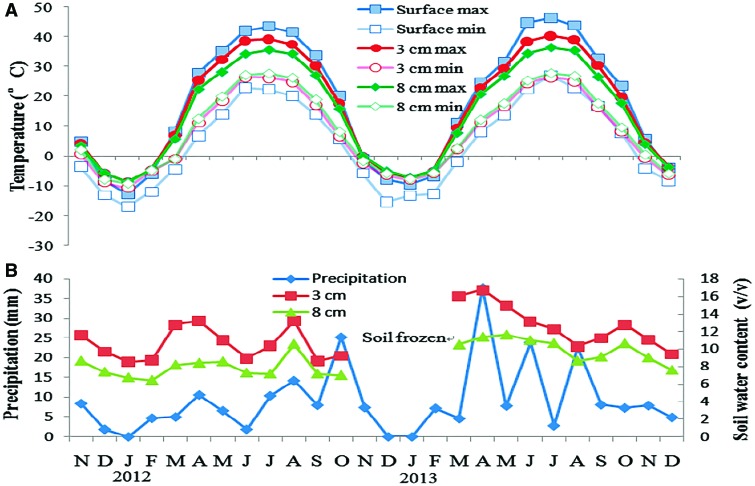
(A) Mean monthly maximum and minimum temperatures at the soil surface and 3 and 8 cm at the field study site of E. songoricum. (B) Precipitation and soil moisture at depths of 3 and 8 cm.

The maximum and minimum temperature at the soil surface exhibited greater fluctuations than those at 3 and 8 cm depths (Fig. 2A). Mean monthly maximum temperatures of the sand surface (41.6–46.3 °C) were the highest in summer (June, July and August) and exceeded maximum temperatures at sand depths of 3 and 8 cm by 3.3–10 °C. In winter (December, January and February), the lowest temperatures at sand depths of 3 and 8 cm were higher than they were on the surface by about 15 °C.

Precipitation was extremely irregular in 2011–13, and soil moisture at a depth of 3 cm was about 10 % higher than it was at 8 cm (Fig. 2B). Fluctuation of soil moisture in different seasons was higher at the sand surface than at burial depths of 3 and 8 cm. From December to February, the sand surface was covered by snow; maximum snow depth was 20 cm.

### Effect of simulated natural temperature and moisture conditions on PY-break

**Figure 3.**
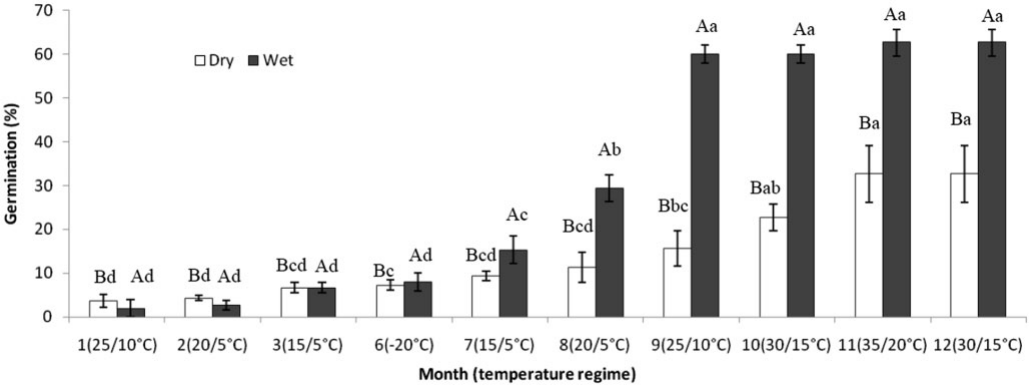
Germination percentages of *E. songoricum* seeds tested in light/dark (12/12 h) at 25/10 °C for 30 days after continuous dry and continuous wet storage during exposure to the simulated sequence of natural temperatures. Different lowercase letters and uppercase letters indicate significant differences in germination percentages of seeds following dry and wet storage at different temperatures for 4 weeks and differences in germination percentages of seeds among dry and wet storage in same treatment temperature (Tukey’ HSD test, *P* < 0.05), respectively. The temperature (−20 °C) was not changed in months 4 and 5, and thus the data analysis were not analyzed.

**Figure 4.**
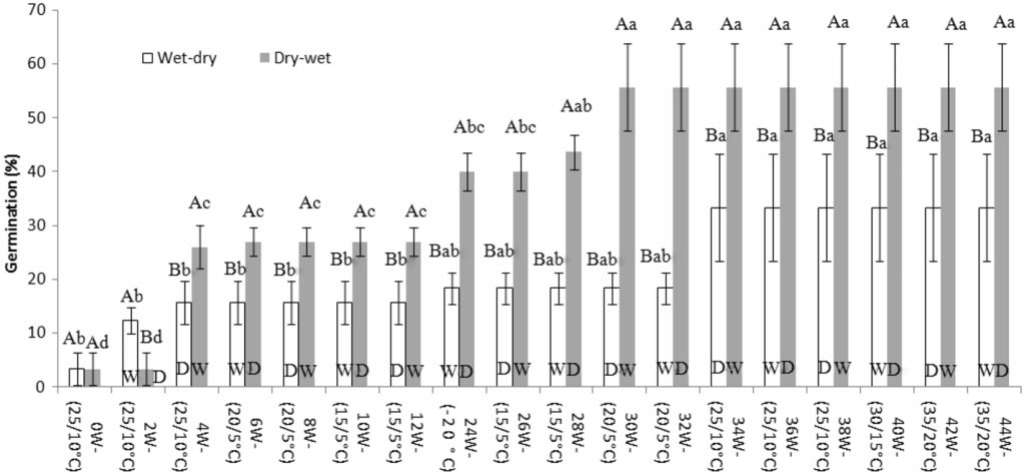
Germination percentages of *E. songoricum* seeds tested in light/dark (12/12 h) at 25/10 °C for 30 days after alternate wet-dry and dry-wet storage during exposure to the simulated sequence of natural temperatures. Different lowercase letters and uppercase letters indicate significant differences in germination percentages of seeds after alternate wet-dry and dry-wet storage for 2 weeks and significant differences in germination percentages of seeds among alternate wet-dry and dry-wet storage in same treatment temperature (Tukey’s HSD test, *P* < 0.05).

Two-way ANOVAs showed that germination percentage differed among moisture conditions (*F*_3,39_ = 150.87, *P* < 0.001), periods of exposure to sequence of temperature regime (*F*_9,39_ = 123.93, *P* < 0.001) and their interaction (*F*_27,39_ = 11.22, *P* < 0.001). For seeds incubated on continuously wet sand, maximum germination (60 %) occurred after seeds had been exposed to the September 2011 through May 2012 temperature regimes and subjected to germination trials. For seeds on continuously dry sand, maximum germination (33 %) occurred in germination trials carried out after seeds had been exposed to the September 2011 through July 2012 temperature regimes (Fig. 3). The percentage of wet-dry and dry-wet seeds in which dormancy was broken was low in seeds exposed to the September through November temperature regimes. Germination percentages of seeds subjected to wet-dry and dry-wet conditions increased (to 33.3 and 58.7 %, respectively) in germination trials after seeds have been exposed to the September through May temperature regimes (Fig. 4).

### Phenology of PY-break in the field

**Figure 5.**
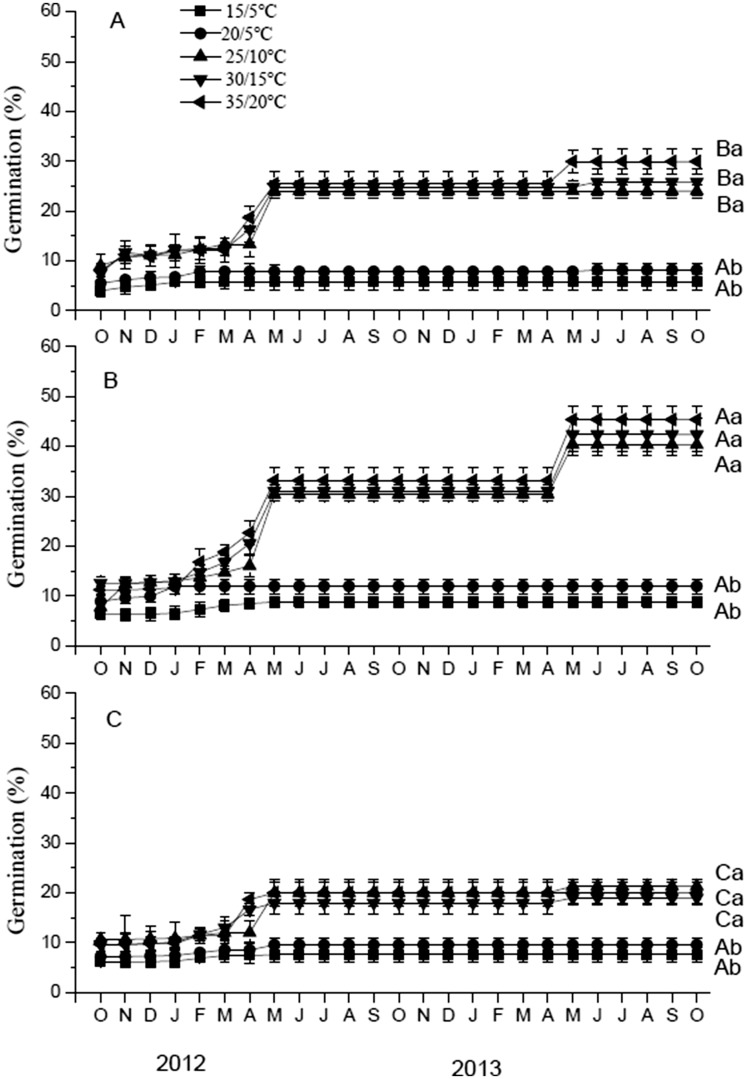
Germination percentages (mean ± SE) of *E. songoricum* seeds incubated on moist filter paper at five temperature regimes in a 12 h photoperiod following 1–25 months on the soil surface (A) and burial at 3 cm (B) and at 8 cm (C). Different lowercase letters and uppercase letters indicate significant differences in final germination percentages of seeds at different temperature regimes at the same depths and significant differences in final germination percentages of seeds among depths in same temperature regimes (Tukey’s HSD test, *P* < 0.05).

Seeds on the soil surface and those buried at 3 and 8 cm exhibited a single annual peak of germination in spring with little or no germination in the other seasons (Fig. 5). For each month of both 2012 and 2013, germination percentages were higher for seeds buried 3 cm than they were for seeds on the soil surface, which in turn were higher than for seeds buried 8 cm. GLM showed that the final germination percentage at each month differed among burial depths (*F*_2, 30_ = 111.837, *P* < 0.001) and temperatures regimes in germination trials (*F*_4, 30_ = 160.108, *P* < 0.001) and their interaction (*F*_8, 30_ = 13.727, *P* < 0.001). Most seeds exhumed in spring germinated at 25/10, 30/15 and 35/20 °C; only a very few seeds germinated at 15/5 or 20/5 °C (Fig. 5). Pearson correlation analysis showed that a significant positive correlation between germination percentage and soil temperature (0cm, *r* = 0.610, *P* = 0.002; 3 cm, *r* = 0.630, *P* = 0.001; 8cm, *r* = 0.559, *P* = 0.004), and unsignificant correlation with soil moisture (0 cm, *r* = 0.308, *P* = 0.143; 3 cm, *r* = 0.281, *P* = 0.184; 8 cm, *r* = 0.295, *P* = 0.162). Partial correlation analysis showed that a significant correlation between germination percentage after 2 years in the field and soil temperature when soil moisture was controlled. Soil moisture was not related to germination percentage when temperature was controlled (Table 1).

**Table 1.** Partial correlation analysis of the relationship between germination percentage and soil temperature and moisture at different burial depths.

		25/10 °C		30/15 °C		35/20 °C	
Depths	Controlling aspects	*r* value	*P* value	*r* value	*P* value	*r* value	*P* value
At surface	soil moisture	0.439	0.036	0.480	0.020	0.556	0.006
	soil temperature	0.088	0.691	0.091	0.679	0.067	0.762
At 3 cm	soil moisture	0.569	0.005	0.589	0.003	0.592	0.003
	soil temperature	0.051	0.819	0.082	0.710	0.089	0.686
At 8 cm	soil moisture	0.444	0.034	0.514	0.012	0.496	0.016
	soil temperature	0.083	0.824	0.049	0.824	−0.020	0.928

## Discussion

Sequence of temperature conditions and moisture had an effect on PY release in *E. songoricum* seeds. In both laboratory and field studies, PY break was highest after seeds had been exposed to autumn plus winter temperatures than it was for those exposed to only autumn temperatures. Simulated conditions in the lab showed dry conditions inhibit PY break, but moisture was not associated to germination percentages of seeds under natural conditions in the field. Further, after seeds had been exposed to winter temperatures in the field they germinated at 25/10, 30/15 and 35/20 °C but not at 15/5 or 20/5 °C (Fig. 5), showed that *E. songoricum* needed late spring or summer temperatures to germinate.

PY-break for seeds *E. songoricum* exhumed after various periods of burial under cold conditions in the field is somewhat similar to that for seeds of *Biserrula pelecinus*, in which a high proportion of seeds softened during the second summer after they have been subjected to winter conditions (Loi *et al.*, 1999). In addition, seeds of several papilionaceous legumes (*Melilotus albus*, *Medicago lupulina*, *Lotus corniculatus* and *Trifolium repens*) incubated at 5 °C (simulated winter conditions) for 8 weeks germinated to high percentages after transfer to spring (15/6 or 20/10 °C) temperatures (Van Assche *et al.*, 2003).

Germination percentage of *E. songoricum* seeds during exposure to the simulated sequence of natural temperature regimes increased after exposure to winter temperatures (−20 °C) for 3 months. Further, either continuously wet or alternately dry-wet were more effective in breaking PY than dry and wet-dry conditions. The difference between wet-dry and dry-wet treatments in the breaking of PY can be related to whether a dry or wet treatment is given first. For example, seeds of *Geranium dissectum* required a wet and then a dry treatment for PY to be broken. If dry came first, PY break was delayed until seeds of *G. dissectum* had received a wet and then a dry treatment (Gama-Arachchige *et al.* 2012).

For *E. songoricum*, seeds that received a dry period first eventually germinated to a higher percentage than those given a wet period first (Fig. 4). Nevertheless, there were increases in germination percentages when seeds were given both treatments: wet to dry (at 25/10, −20 and 25/10 °C) and dry to wet (at 25/10, −20 and 20/5 °C). Our results showing that wet conditions at low temperatures promote breaking of PY are in contrast to the dry heat (40, 60 or 80 °C) required to break PY in seeds in Mediterranean climates (Morrison *et al.*, 1998) and the wet heat requirement for species in a tropical climate (Van Klinken *et al.* 2006). Regardless of the wetting and drying regime, incubation of *E. songoricum* seeds at 30/15 and 35/20 °C did not increase germination. Spring dormancy-break in seeds of *E. songoricum* is in contrast to that of seeds of the winter annuals *Geranium carolinianum, G. dissectum* (Gama-Arachchige *et al.* 2012), *Medicago polymorpha* (Taylor and Ewing 1996) and *Trifolium glomeratum* (Smith *et al.* 1996) that become permeable in autumn, after being exposed to summer environmental conditions.

The higher PY-break and germination percentages of *E. songoricum* seeds at 3 than at 0 and 8 cm is similar to results of some, but not all, studies in which water-impermeable seeds were buried at different depths in the field (Loi *et al* 1999; Zeng *et al.* 2005; Van Klinken *et al.* 2006). Variation in temperature and substrate moisture content among different burial periods and depths are considered key factors that influence PY-breaking in natural conditions (Norman *et al.* 2002; Van Klinken *et al.* 2006, 2008; Hu *et al.* 2009). However, substrate moisture does not seem to play an important role in modulating PY- break of *E. songoricum* seeds under natural conditions. Germination percentages of *E. songoricum* seeds exhumed from 3 cm soil depth in both years of our study was higher at 25/10 °C than that for seeds on the sand surface or at a depth of 8 cm. The higher dormancy break at 3 than at 8 cm may be related to the higher temperature and/or higher amplitude (maximum and minimum) of daily temperature fluctuation at 3 cm. Although both temperature and amplitude of temperature fluctuation were higher on the soil surface than at 3 cm, low sand-moisture content may have been responsible for the lower germination percentages of seeds on the surface. Loss of viability of seeds in the field could have affected seed germination, but we do not think that is the case. Liu *et al.* (2011) showed that 99, 98 and 99 % of *E. songoricum* seeds were viable after 7 months on the surface and at 3 and 8 cm, respectively. Thus, most seeds that become buried to a depth of 8 cm or deeper may be viable and remain dormant until they are returned to shallow sand depths by disturbance or by wind removing some of the sand covering them. In its cold desert sand dune habitat, *E. songoricum* seeds are dispersed in late summer and early autumn. However, moisture content of the sand at depths of 3 and 8 cm is adequate (>5 %) for seed germination throughout the growing season, because the sand-moisture threshold for seed germination and seedling survival of *E. songoricum* is only 2.0 % (Liu *et al.* 2011). Thus, there is enough moisture in the sand at 3 and 8 cm for nondormant seeds of *E. songoricum* to germinate in the summer and autumn. Lack of germination of *E. songoricum* seeds at 3 and 8 cm in these seasons indicates that PY is not broken. Thus, dormancy break did not occur until seeds were exposed to winter/early spring conditions, thereby ensuring that seeds can germinate in mid- to late spring, when conditions are best for seedling growth. We obtained relatively high germination percentages after exposure to simulated winter temperature (−20 °C). However, it is not known if the temperature regime of September through November must proceed the low winter (−20 °C) temperature in order for the latter to be effective in the dormancy-breaking protocol of *E. songoricum* seeds.

Only a portion of the seeds of *E. songoricum* becomes permeable and germinates the first spring. A consistent pattern of spring emergence of seedlings has been reported for several other non-winter annual species of Fabaceae (Roberts and Boddrell 1985; Baskin and Baskin 2014). In our study, a small proportion of *E. songoricum* seeds germinated in the spring, suggesting that seeds in a seed cohort differ in amount of heat and/or cold required to break PY. Presumably, it takes several years for seeds in a seed cohort of *E. songoricum* to become water-permeable, as has been documented for other species of Fabaceae (Baskin and Baskin 2014).

## Conclusions

*E. songoricum* exhibits seasonal periodicity of seed germination in its cold desert sand dune habitat. PY-break and germination are controlled by the sequence of winter–spring temperature and sand moisture conditions. Thus, the seasonal cycle of natural temperature and moisture conditions in the habitat of *E. songaricum*, together with the seed dormancy-breaking requirements of this species, are such that germination occurs only in spring. To our knowledge this is the first study to document seasonal periodicity in seed germination in a cold desert species with PY and to identify the mechanism (at the whole-seed level) of its occurrence.
